# A global dynamic runoff application and dataset based on the assimilation of GPM, SMAP, and GCN250 curve number datasets

**DOI:** 10.1038/s41597-022-01834-0

**Published:** 2022-11-16

**Authors:** Lara H. Sujud, Hadi H. Jaafar

**Affiliations:** Department of Agriculture, American University of Beirut, Beirut, Lebanon

**Keywords:** Hydrology, Hydrology

## Abstract

Runoff modelling is a crucial element in hydrologic sciences. However, a global runoff database is not currently available at a resolution higher than 0.1°. We use the recently developed Global Curve Number dataset (GCN250) to develop a dynamic runoff application (2015 – present) and that can be accessed via a Google Earth Engine application. We also provide a global mean monthly runoff dataset for April 2015-2021 in GeoTIFF format at a 250-meter resolution. We utilize soil moisture and GPM rainfall to dynamically retrieve the appropriate curve number and generate the corresponding runoff anywhere on Earth. Mean annual global runoff ratio results for 2021 were comparable to the runoff ratio from the Global Land Data Assimilation System (0.079 vs. 0.077, respectively). Mean annual global runoff from GCN and GLDAS were within 11% each other for 2020–2021 (0.18 vs. 0.16 mm/day, respectively). The GCN250 runoff application and the dataset are useful for many water applications such hydrologic design, land management, water resources management, and flood risk assessment.

## Abstract (summary)

**Table:** 

Measurement(s)	runoff
Technology Type(s)	Natural Resources Conservation Service Curve Number Method
Factor Type(s)	Curve Number • Soil Moisture • Rainfall
Sample Characteristic - Environment	land

## Background & Summary

Accurate estimation of surface runoff is attracting increasing interest in hydrologic sciences due to its significance in water resources management. Surface and subsurface runoff quantification has many applications in the field of hydrology. This quantification is valuable for basin water monitoring, hydrologic modelling and design, water infrastructure design, and flood risk assessment, groundwater recharge, among other applications^1^. Acquiring runoff data from gauging stations is a challenging task that often requires high installation and maintenance costs^2^. The availability of global, continuous, and more importantly reliable and easily accessible runoff data is of increasing relevance and of high value.

The Natural Resources Conservation Service Curve Number method (NRCS-CN), previously known as the Soil Conservation Service-Curve Number (SCS-CN) method, is a commonly used model in estimating rainfall runoff ^3^. The method was developed by the USDA SCS in 1954 and has gained popularity since then due to its simplicity, reliability, credibility and its responsiveness to soil type, land use, surface condition and antecedent condition^4^. Originally, the purpose of NRCS method was to obtain runoff estimates for agricultural watersheds from storm rainfall events in the USA^5^. However, due to its convenience, water resources practitioners have adopted its usage in multiple regions and land uses^6–13^. In addition to its application in rainfall-runoff modelling, the NRCS-CN method has been successfully applied in urban hydrology^14–16^, water quality^17–19^, rainwater harvesting^3,20–22^, subsurface flow estimation^23–26^, and estimation of evapotranspiration (ET)^27–29^. This widespread implementation of the NRCS-CN method proves its prominence in hydrologic applications. Many techniques to estimate global surface runoff are computationally demanding and time consuming. Satellite observations, acquired in near-real time, can provide reliable surface runoff estimates at a hydrologically relevant spatial resolution^30^. In this context, this paper proposes a robust approach to quantify surface runoff – at the pixel, watershed, regional, and global scales – in near-real time at a 250 m resolution exploiting the NRCS-CN method. The generation of a near real-time, terrestrial time-series runoff is of immense importance in a better quantification of surface runoff and flood events, especially in ungauged watersheds. Applications of the runoff generator can help in agricultural planning, hydrological engineering, flood forecasting and monitoring water resources.

**Fig. 1:**
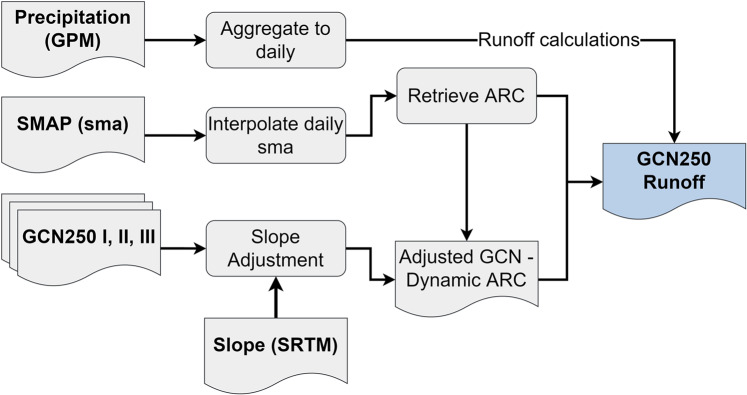
Workflow of generating global runoff data in Google Earth Engine.

Recently, a global dataset for Curve numbers at the 250 m resolution (GCN250) has been published and is publicly available^1^. GCN250 has been validated against runoff generated from GLDAS (Global Land Data Assimilation System) but not against gauge runoff data. The dataset can benefit from Google Earth Engine (GEE)^31^ as the leading planetary-scale geospatial analysis platform by first adjusting the curve number by slope and second validating it against runoff data using remotely-sensed real-time estimates of precipitation. Such an application will yield a global runoff generator building on the GCN250 dataset, which accounts for wet, average, and dry antecedent runoff conditions. Motivated by the generation of GCN250m data, this paper attempts to create a synergistic global runoff generator (a GEE app) exploiting the surface soil moisture data product (SMAP)^32^, which became available in 2015. We use SMAP surface soil moisture anomalies and remotely sensed precipitation data from the Global Precipitation Measurement Mission (GPM) in near real-time to dynamically retrieve the appropriate curve number (wet-average-dry) from the GCN250m dataset based on the antecedent runoff condition (ARC, previously known as antecedent moisture condition). ARC is an indication of the runoff potential before a storm occurs, and it accounts for the variation in CN caused by different storms^4^. We developed a readily usable method to estimate global runoff using GEE (Fig. 1). The developed runoff generator estimates surface runoff using the GCN250m, SMAP surface soil moisture anomalies, precipitation from GPM^33^, and elevation data from the Shuttle Radar Topography Mission (SRTM)^34^. The tool provides a simple and reliable solution for runoff generation, which will be of value and interest to the scientific community because it can be directly used in generating time-series runoff for any watershed (given the availability of a consistent SMAP soil moisture product from April 1, 2015, until near-real time, and rainfall data from GPM). We further validated our results with daily discharge data from USGS (2015–2020), and annual runoff data from NASA’s Global Land Data Assimilation System (GLDAS)^35^ and from the European Centre for Medium-Range Weather Forecasts (ECMWF)^36^.

## Methods

### Datasets acquisition

We used several satellite sensors to generate runoff: the recently developed GCN250m global curve number dataset, the Soil Moisture Active Passive (SMAP) mission, the Shuttle Radar Topography Mission (SRTM) for elevation and the Global Precipitation Measurement Mission (GPM). The publicly available GCN250m dataset consists of three curve number maps accounting for three antecedent runoff conditions: dry, average, and wet at a 250 m spatial resolution^1^. We imported the GCN250m dataset into GEE assets to use it in runoff calculations. The SMAP mission utilizes L-band radar and radiometer instruments and provides direct observations of global soil moisture every two to three days at a 9 km spatial resolution^32^. From the SMAP mission, we use the surface soil-moisture anomalies (ssma) determined from the climatology of the day of interest. SMAP was accessible on GEE with a one-week delay. We obtain daily precipitation (in mm) from GPM 3-hourly satellite mission available on GEE in near-real time. All datasets were prepared using GEE. Streamflow data from the U.S. Geological Survey (USGS) Surface-Water Data for the USA were obtained for runoff validation. Results were compared against USGS runoff, European Centre for Medium-Range Weather Forecasts (ECMWF) runoff and Global Land Data Assimilation System (GLDAS) runoff.

### Runoff estimation and adjustments

#### The NRCS-CN method

From daily rainfall, we estimate rainfall-runoff using the original NRCS-CN runoff equation^37^:

1 $$Q=\frac{{(P-{I}_{a})}^{2}}{(P+{I}_{a})+S}\quad P > {{\rm{I}}}_{{\rm{a}}},\quad {\rm{else}}\;Q=0$$

Where Q, P, and I_a_, S = runoff depth, rainfall depth, initial abstraction, and maximum potential retention, respectively. Initial abstraction, which consists of infiltration, surface depression storage and canopy interception during the early parts of a storm^37^, was assumed to be a function of the potential maximum retention (I_a_ = λS). which consists of infiltration, surface depression storage and canopy interception during the early parts of a storm^37^. According to the NRCS, it is recommended that λ = 0.2 for widespread use. We use λ = 0.2 because the original curve number calculation was based on a λ = 0.2. The potential maximum retention, S, can be determined from the curve number dataset using the equation:

2 $$S=25.4\left(\frac{1000}{CN}-10\right)\quad \quad {\rm{For}}\;{\rm{S}}\;{\rm{in}}\;{\rm{mm}}$$

Since S is in millimeters, then P and Q are also expressed in millimeters.

#### Slope adjustments

Terrain slope can affect runoff prediction by reducing infiltration and recession time. Here we, adjust CN values for slope. The average CN map obtained from GCN250m is appropriate for slopes up to 5%. We adjust the CN map for slope based on the Sharpley-Williams method^38^ presented in Eq. (3).

3 $$C{N}_{2\alpha }=\frac{1}{3}\left(C{N}_{3}-C{N}_{2}\right)\left(1-2{e}^{-13.86\alpha }\right)+C{N}_{2}$$

Where CN_2α_ is slope-adjusted CN for average ARC, CN_3_ is CN for wet ARC, and CN_2_ stands for CN for average ARC, and α is terrain slope in m/m.

After adjusting the average ARC condition CN map, we use the table in the National Engineering Handbook – Part 630 Hydrology to obtain values of CN_1_ and CN_3_. We apply the below equations^37,38^ to compute CN_1_ and CN_3_ from slope-adjusted CN_2_:

4 $$C{N}_{1}=C{N}_{2\alpha }-\frac{20\left(100-C{N}_{2\alpha }\right)}{100-C{N}_{2\alpha }+{e}^{\left[2.53-0.0636\left(100-C{N}_{2\alpha }\right)\right]}}$$

5 $$C{N}_{3}=C{N}_{2\alpha }\times {e}^{\left[0.00673\left(100-C{N}_{2\alpha }\right)\right]}$$

#### Accounting for antecedent runoff condition

The antecedent runoff condition (ARC) is affected by rainfall intensity and duration, soil moisture conditions, vegetation cover, stage of growth and temperature^1^. The CN values vary depending on ARC. We use surface soil-moisture anomalies (ssma) obtained from SMAP to retrieve the appropriate curve number based on the ARC. Since SMAP data is available every two to three days, we interpolate the anomaly values on days where data is missing. We retrieve the appropriate CN value using an “if – then” algorithm:

If ssma ≤−1.5, then CN = CN_1_ (dry ARC);

If ssma ≥1.5, then CN = CN_3_ (wet ARC);

Otherwise, CN = CN_2_ (average ARC)

#### Development of the app and upstream watershed delineation

We develop an application that provides users with on-click pixel runoff at 250 m resolution and a time-series of CN-based runoff over point of interest or watershed of interest. Upstream basins are delineated automatically within the app using the HydroBASINS product. Three columns from the attribute table of the HydroBASINS product are used: the “main_bas”, the “hybas_id” and the “next_down” columns. Sub-basins that share the same main basin (i.e. “main_bas” value) are filtered based on point of interest. The “next_down” column represents the ID of the next downstream polygon. We use it along with the “hybas_id” column to navigate downstream using a look-up dictionary. The resulting output is the upstream basin of the point of interest. The users have the option to either draw their point/basin of interest or use the upstream basin generation method in the app (which is based on the topological concept of the Pfafstetter coding system^39^). The app provides the users with the option to export both the time-series runoff as csv and the delineated upstream basin as a shapefile.

**Fig. 2:**
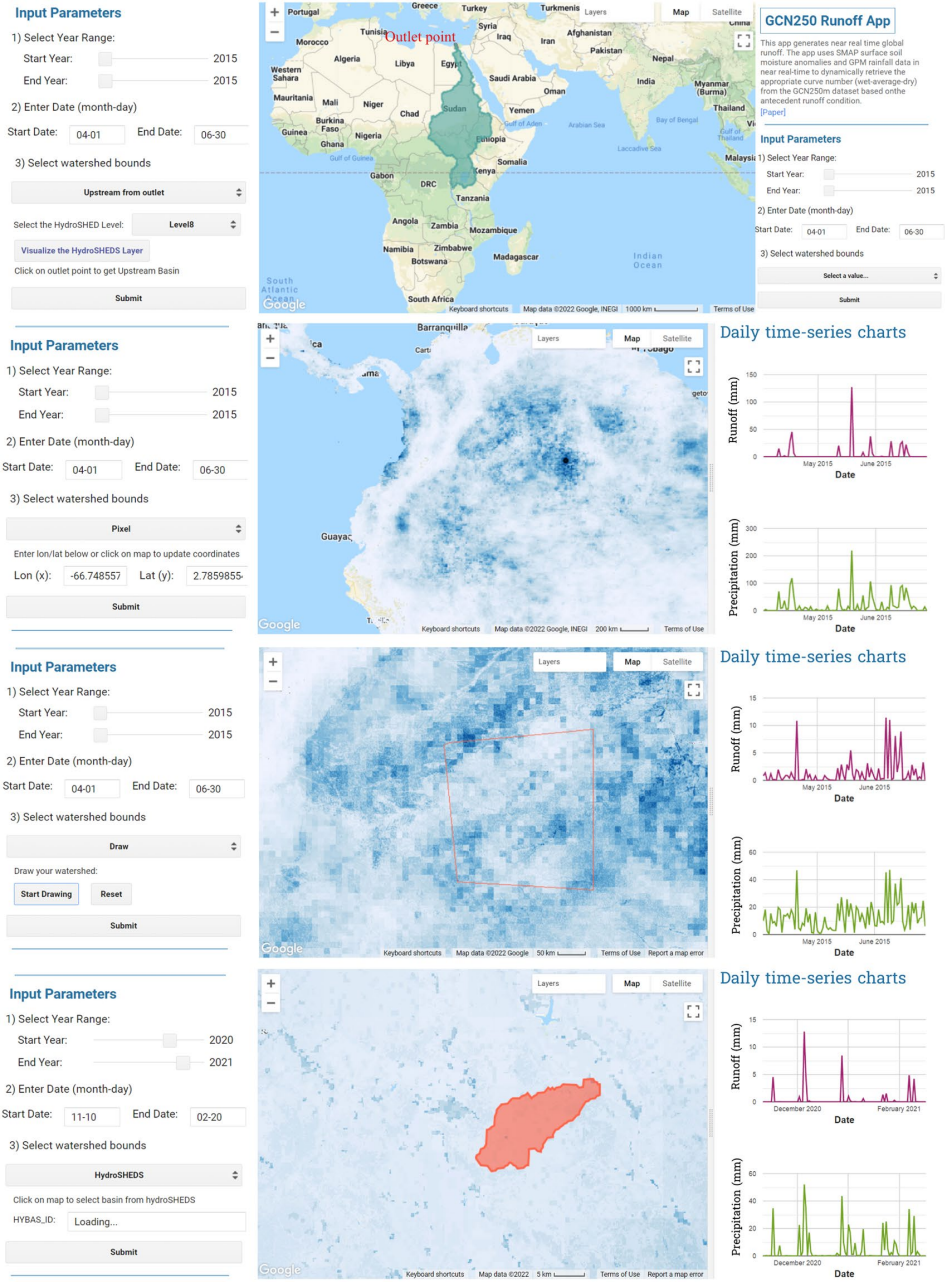
GCN250 Runoff application interface in Google Earth Engine.

The application of Geographic Information System (GIS) in hydrologic modeling and water resources management requires large data and computing resources. The developed runoff app takes advantage of GEE’s powerful cloud computing environment to process big data in minutes thus overcoming the issue of limited computing capacity and saving time. The global runoff generator presented herein is based on the assimilation of three global datasets: the GCN250 curve number dataset, the GPM rainfall product, and the soil moisture product (SMAP). As an application in GEE, the GCN250 Runoff app can be used by users without prior knowledge of JavaScript, and without the need to import or store any data required for runoff calculation. The major capability of the GCN250 Runoff app is that a time-series of rainfall-runoff for any watershed can be generated for an extended period of record instantaneously which reduces the time and effort needed in generating runoff as compared to other methods of runoff generation. This would reduce the workload of hydrologists and researchers who work on hydrologic modeling and runoff generation using the NRCS-CN model. Time-series daily runoff values are plotted over either a point, a basin selected from HydroSHEDS basins collection, or a generated upstream basin from an outlet point of interest (Fig. 2). Users can use the HydroBasin Tool to choose the HydroBASIN level of interest, and the Upstream Basin Tool to delineate the upstream basin of a user-defined outlet point.

The output of the app includes mapping runoff, rainfall, and curve number values anywhere on land, exporting time-series values as csv, charting time-series (Fig. 2), and exporting the upstream basin as a shapefile. Other uses of the app include exploring runoff and rainfall time-series for different watersheds globally, visualizing daily runoff, rainfall and curve number values based on change in soil moisture anomalies, and exporting the time-series of daily runoff.

Figure 2 shows the GCN250 Runoff app in Google Earth Engine. Designed to automate the process of hydrologic analysis, the purpose of the app is to facilitate the generation of rainfall-runoff using the curve number method. Obtaining time-series rainfall-runoff values on-click makes hydrologic modeling more efficient and effective. Other than generating runoff values, users can simultaneously observe the response of soil moisture anomalies, curve number values, and runoff to changes in rainfall over a pixel-level (at 250 m resolution) and watershed level, globally. The app is easy to use. It only requires two inputs: a period of study (start and end date) and a watershed of interest (or point of interest). No prior knowledge of GEE or JavaScript is required to run the app. All the layers and datasets that are needed for runoff calculation are available as GEE datasets and need not to be imported by the user. The Antecedent Runoff Condition (ARC) is automatically selected based on the surface soil moisture anomalies values per pixel.

## Data Records

**Fig. 3:**
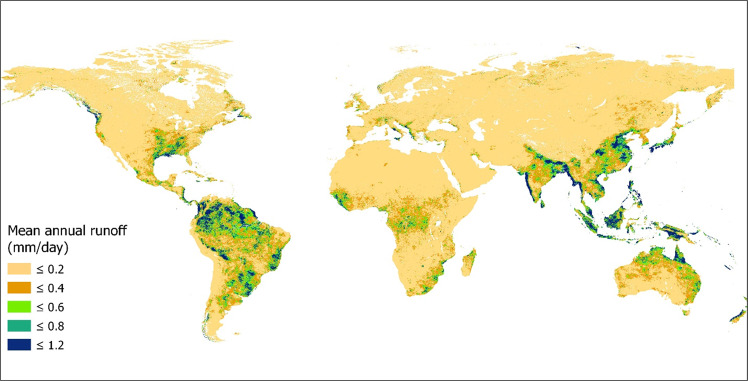
Generated annual mean runoff for 2021 using the GCN250 Runoff application.

The 2021 mean annual GCN250 Runoff dataset at 250 m resolution is publicly available in the Figshare database (10.6084/m9.figshare.19596157)^40^. The product is stored in GeoTiff format at 7.5 arc-second (~250 m spatial resolution) using the World Geodetic System 1984 (WGS84) datum geographic coordinate system. All data can be generated by any user using the publicly available application and can be exported in csv format in Google Chrome (https://jaafarhadi.users.earthengine.app/view/runoff-from-gcn250). The monthly mean GCN Runoff data for 2015–2021 is hosted on GEE as an *Image Collection (*https://bit.ly/3T321KH). The pixel values need to be multiplied by 0.001 to obtain the runoff in mm. Figure 3 shows the GCN250 Runoff dataset for 2021.

## Technical Validation

The generated runoff prediction accuracy was assessed by comparing it to the observed USGS streamflow data. It remains a challenge to directly compare runoff observations from USGS to our CN generated runoff. Pixel-based generated runoff provides information on the spatial distribution of runoff within the watersheds which is not the case when using the observed streamflow data from USGS. However, it is crucial that generated runoff is evaluated using the observed runoff. To quantitatively evaluate the generated rainfall-runoff accuracy, we used statistical performance indices in combination with graphical performance measures. The combination of quantitative statistical indices and graphical performance measures provides robust model accuracy and performance assessment^41^. The most common statistical indices were used: the Coefficient of determination (R^2^), Root Mean Square Error (RMSE), and Nash-Sutcliffe efficiency (NSE). We used time-series graphs for visual comparison and to better observe and identify model bias (i.e., when and where the model is performing inadequately).

The validation results show that the GCN250 Runoff app provides runoff data that correlates well with ground-truth discharge data from USGS. Further development of the app can be performed by integrating more functions and equations or applying automatic calibration to the model.

### Runoff Validation with USGS discharge data

**Fig. 4:**
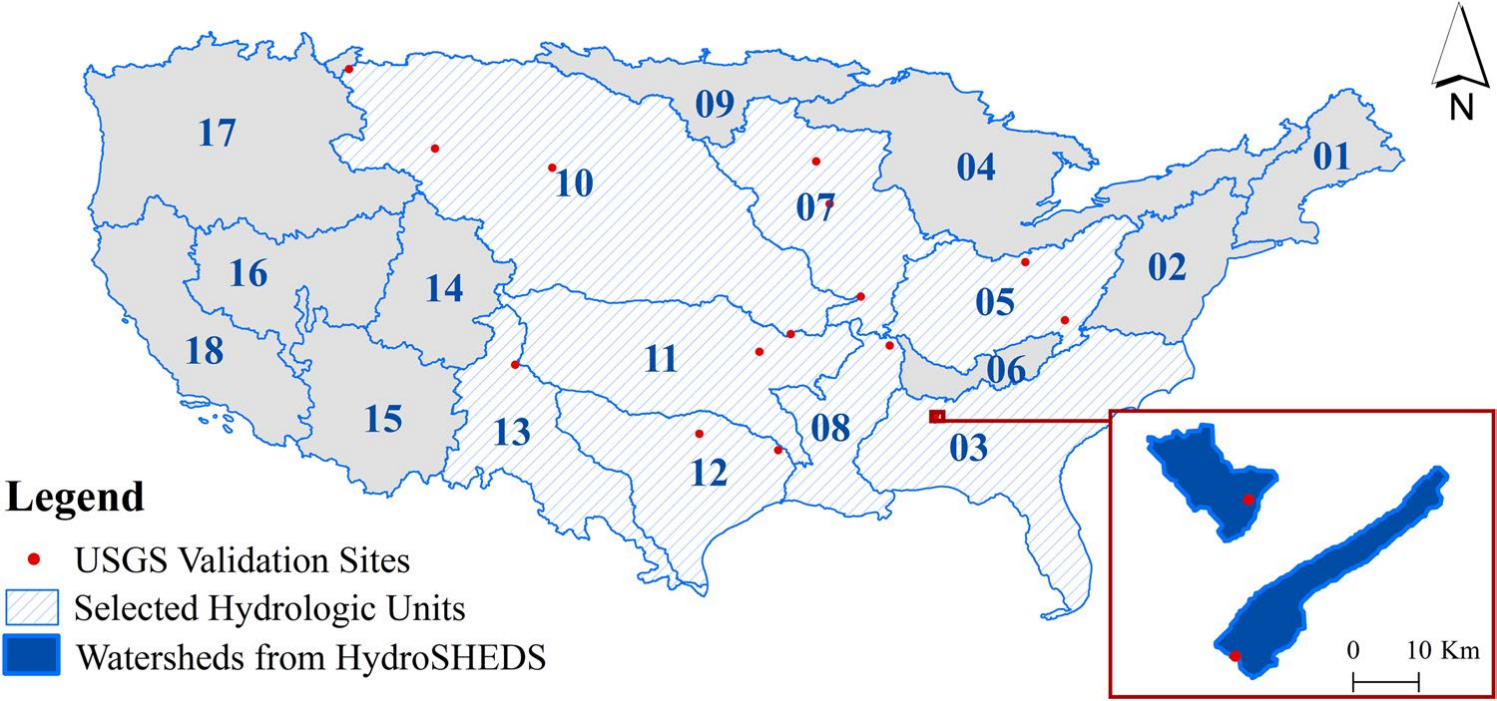
Selected watershed locations used for validation.

We validated the generated runoff results against 16 watersheds in the US from 8 hydrologic units having 4 different climatic characteristics for period 2015–2020. The USGS national database includes a wide range of hydrologic data that is freely available to the public through their web interface (http://waterdata.usgs.gov). Sixteen sites were selected for validation (Fig. 4).

We aggregated the daily runoff data, both observed and predicted, into monthly means to study the correlation between the observed and predicted runoff values (in mm/month). Watersheds in hydrologic unit 11 showed the largest correlation (goodness-of-fit, R^2^ = 0.8), along with one watershed from hydrologic unit 12 (R^2^ = 0.8). There was a good correlation (R^2^ > 0.5) in seven watersheds coming from different hydrologic units. Moderate correlation was found in five watersheds (R^2^ > 0.375), and weak correlation in one watershed (R^2^ = 0.2). As shown in Table 1, goodness-of-fit (R^2^) varies between watersheds within the same hydrologic unit which we believe is due to difference in specific watershed characteristics such as morphologic and vegetation factors.

**Table 1:** Selected sites for validation and their corresponding hydrologic unit number, goodness of fit and root (R^2^) mean square error (RMSE).

Site Name	HUC	R^2^	RMSE (mm/month)
Rio Mora near Terrero	13	0.561	0.01
Crow Creek Near Beulah	10	0.694	0.02
Willow Creek near Boyd	10	0.468	0.04
NF Milk River ab St. Mary canal near Browning	10	0.497	0.07
Bayou De Chien Near Clinton	8	0.611	0.15
Eau Galle River at Spring Valley	7	0.388	0.18
Piney Creek at Raleigh	5	0.375	0.2
Alum Creek near Kilbourne	5	0.213	0.21
Bayou Grand Cane near Stanley	12	0.599	0.23
Kickapoo Ck at Onalaska	12	0.815	0.26
Wilson Creek near Brookline	11	0.841	0.29
Otter Creek at Elgin	7	0.479	0.31
Shades Creek near Greenwood	3	0.673	0.31
Coldwater Creek near Blackjack	10	0.737	0.32
Beaty Creek near Jay	11	0.817	0.32
Village Creek near Docena	3	0.671	0.36

### Performance by hydrologic units, climate, and soil characteristics

**Fig. 5:**
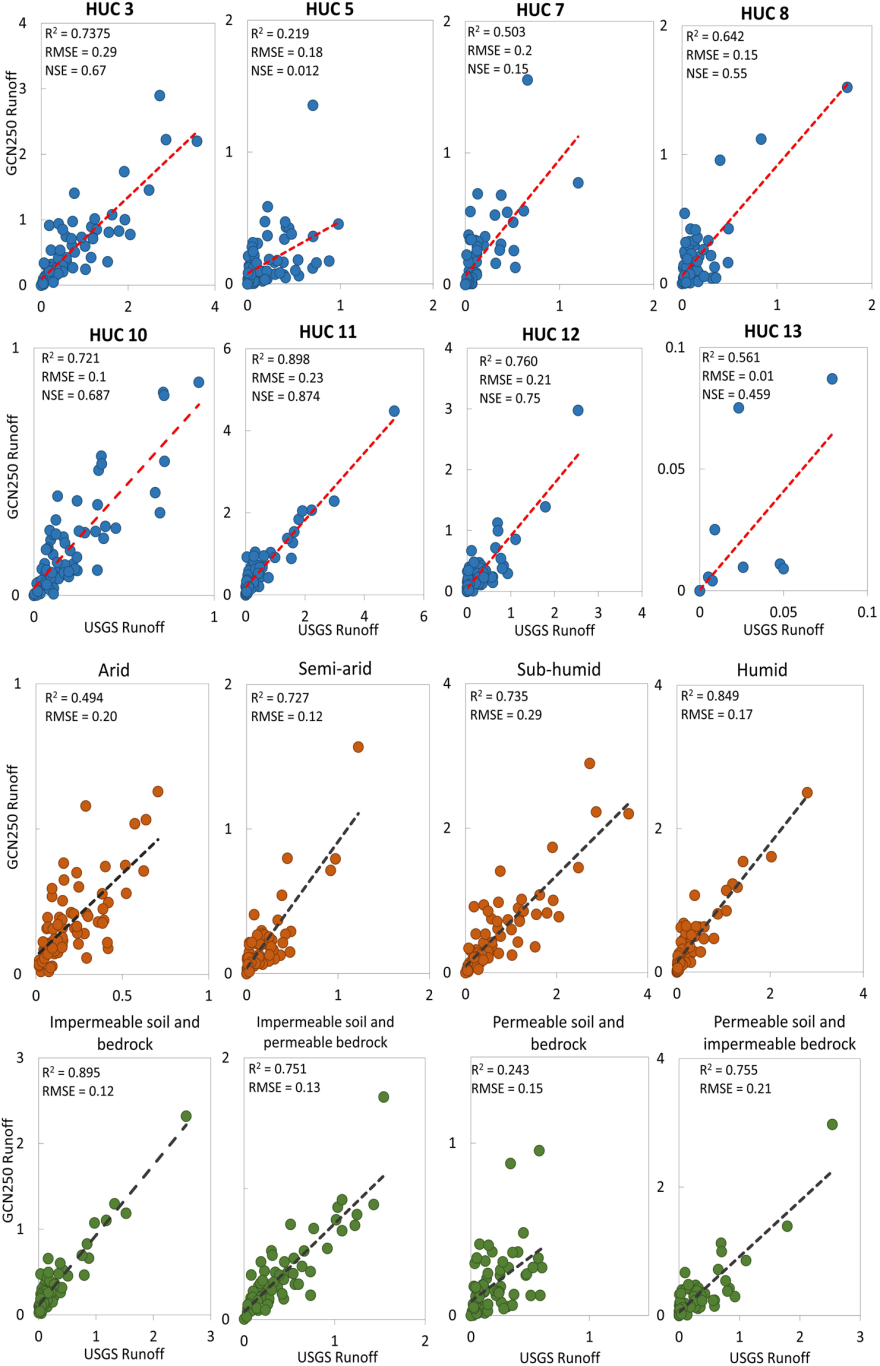
Comparison of USGS observed runoff and GCN250 generated runoff (mm/month) (**a**) Correlation results grouped by hydrologic unit code (HUC); (**b**) Correlation results grouped by climatic characteristics and soil permeability.

Results showed strong correlation for watersheds in hydrologic units 3, 10, 11 and 12, a moderate correlation for watersheds in hydrologic units 8, 13 and 7, and a weak correlation for watersheds in hydrologic unit 5. The strongest correlation (R^2^ = 0.898) was found in watersheds of hydrologic unit 11, characterized by impermeable soils and bedrock and having a humid climate. Watersheds in hydrologic unit 12 characterized by permeable soils, impermeable bedrock and a semi-arid climate showed a strong correlation ((R^2^ = 0.76) too. A strong correlation (R^2^ = 0.7) was found in watersheds of hydrologic units 3 (sub-humid climate) and 10 (arid climate), characterized by impermeable soils and permeable bedrock. A good correlation was found in watersheds of hydrologic units 8, and 13 characterized by impermeable soils and bedrock and a semi-arid climate. Watersheds in hydrologic unit 7 characterized by a humid climate and permeable soils and bedrock showed a moderate correlation (R^2^ = 0.5). There was a weak correlation (R^2^ = 0.2) in hydrologic unit 5 characterized by permeable soils and bedrock and an arid climate. The Root Mean Square Error (RMSE) ranged between 0.01 (HUC 13) and 0.29 (HUC3). Similarly, NSE values ranged between 0.01 (HUC5) and 0.87 (HUC 11). The variations in R^2^, RMSE, and NSE for the different hydrologic units are shown in Fig. 5a.

Climatic factors, such as rainfall and temperature, have a profound influence on runoff and groundwater. Thus, we group watersheds into four climate zones (Arid, Humid, Semi-arid, and sub-humid) to study how well the model works under different climatic characteristics (Fig. 5b). Results showed there exists a strong correlation in humid climates (R^2^ = 0.85, RMSE = 0.17, NSE = 0.82), followed by semi-arid (R^2^ = 0.73, RMSE = 0.12, NSE = 0.697) and sub-humid climates (R^2^ = 0.73, RMSE = 0.29, NSE = 0.673). The weakest correlation was observed in watersheds of Arid climates (R^2^ = 0.49, RMSE = 0.1, NSE = 0.456).

Knowing that soil permeability is among the physical features that affect runoff, we divide the watersheds based on soils and bedrock permeability into four groups (Fig. 5b). Group 1: watersheds of impermeable soils and bedrock, group 2: watersheds of impermeable soils but permeable bedrock, group 3: watersheds of permeable soils but impermeable bedrock, and group 4: watersheds with permeable soils and bedrock. Results showed that watersheds from group 1 had the highest R^2^, NSE and lowest RMSE value (R^2^ = 0.89, RMSE = 0.12, NSE = 0.868). Similarly, watersheds of groups 2 and 3 showed strong correlation (R^2^ = 0.75). There was a weak correlation in watersheds of permeable soils and bedrock (R^2^ = 0.243).

We observe how watershed area size and slope percent affects correlation results. The predicted runoff values show good correlation with watersheds of different area sizes, with the lowest correlation (R^2^ = 0.6) for area size less than 128 sq. km, and the greatest correlation was noticed for watersheds of area size between 128–140 sq. km. Runoff results from watersheds of higher slope percent (>7%) showed lower agreement (lower R^2^) with USGS observed runoff data as compared to watersheds with slope percent 2.5–7% (R^2^ > = 0.65).

### Validation with GLDAS and ECMWF Runoff

**Fig. 6:**
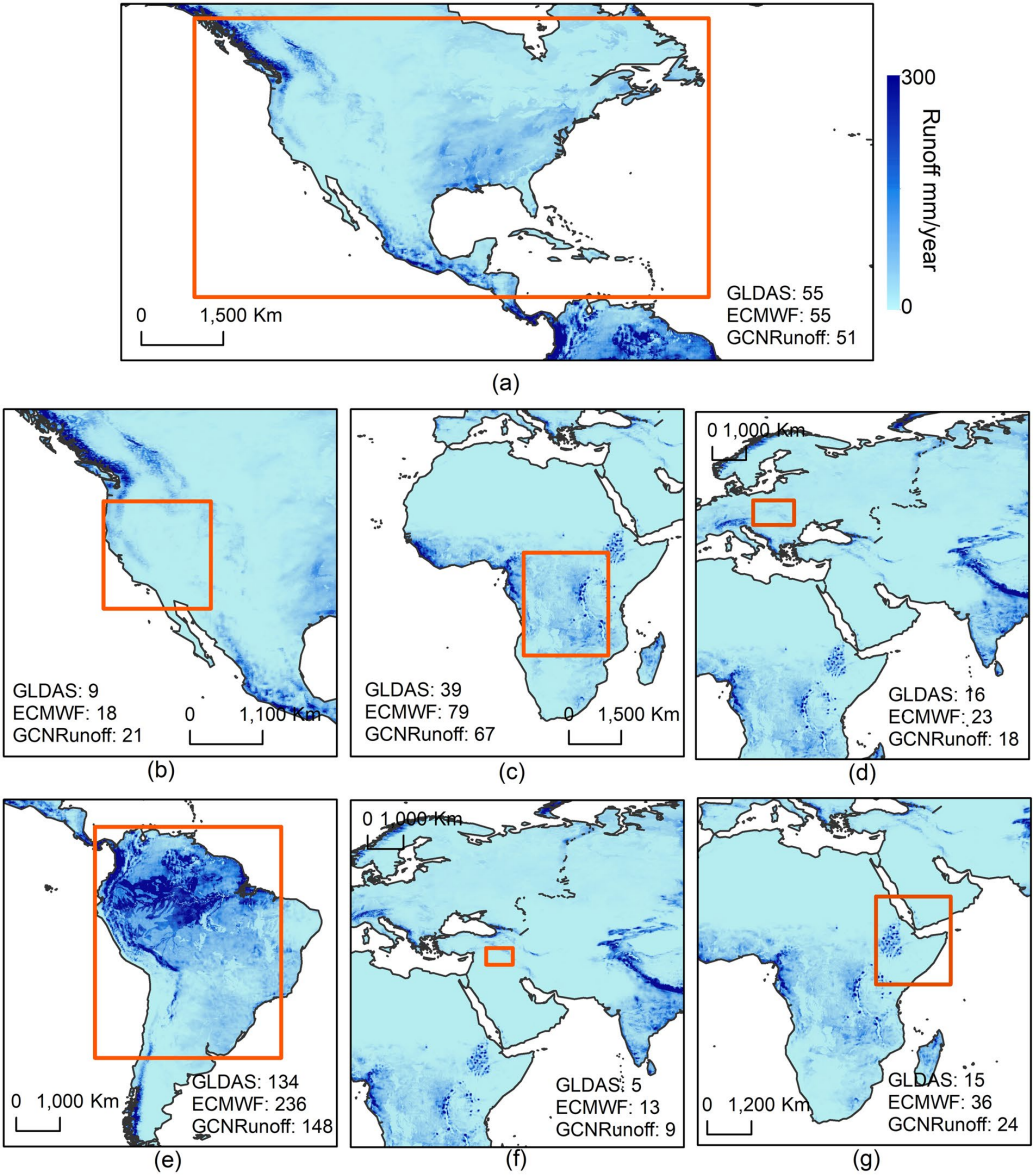
Comparison of average annual runoff (mm/year) for 2021 obtained from GLDAS, ECMWF and GCN250 Runoff for (**a**) United States, (**b**) Sacramento, (**c**) Congo, (**d**) Danube, (**e**) Amazon, (**f**) Euphrates and Tigris, and (**g**) East Africa. As shown in the figure, the GCN250 average annual runoff (mmm/year) lies between average annual runoff from GLDAS and ECMWF, except for (**a**) where it is slightly lower than GLDAS and ECMWF (51 mm/year compared to 55 mm/year), and (**b**) where it is slightly higher (21 mm/year).

**Fig. 7:**
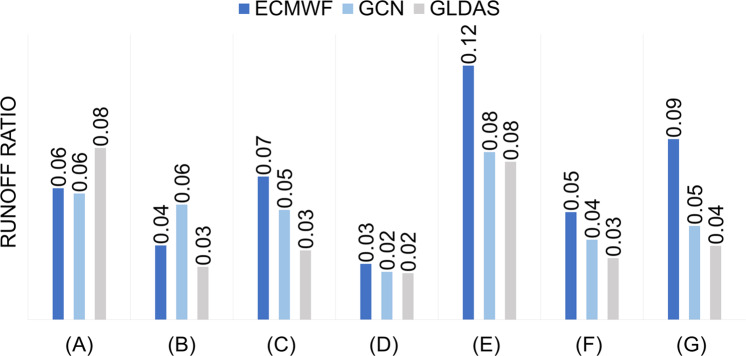
Mean annual runoff ratios of 2021 obtained from ECMWF, GCN Runoff and GLDAS for (**a**) United States, (**b**) Sacramento, (**c**) Congo, (**d**) Danube, (**e**) Amazon, (**f**) Euphrates and Tigris, and (**g**) East Africa.

We further validate the GCN250 Runoff dataset (average runoff for 2021) against runoff from GLDAS and ECMWF for the following regions: the US excluding Alaska, Sacramento (USA), Congo (Africa), Danube (Europe), Amazon (South America), Euphrates and Tigris (Syria, Iraq, Turkey), and part of East Africa (Fig. 6). We also compare the runoff ratios (average runoff/average rainfall) for the same regions (Fig. 7). Average annual runoff ratios were generated using the average annual rainfall (mm/day) from GPM (for GCN250 runoff ratio), GLDAS rain (for GLDAS runoff) and ECMWF precipitation (for ECMWF runoff ratio). Results show a good agreement between runoff ratios from ECMWF and GCN250 runoff ratios in the United States (Fig. 7a). For Congo, Euphrates and Tigris, and East Africa regions, results show that runoff ratios from GCN250 lie between runoff ratios from GLDAS and ECMWF with ECMWF runoff ratios having larger values. For the Amazon and Danube basins, the runoff ratios from GCN250 and GLDAS show good agreement both having a smaller runoff ratio as compared to ECMWF runoff ratios.

We also estimate the global runoff ratios of 2021. The mean global runoff ratio from GCN Runoff was 7.9% compared to 7.7% from GLDAS. The estimated global mean runoff ratio excluded areas of high latitudes (up to the latitude band 60°N-S).

The herein developed runoff app is a tool that makes runoff estimation faster and easier. Moreover, both the app and the generated global curve number runoff product are useful for several applications when combined with other data products. For example, users may want to utilize the output of the app (or the generated runoff product) in water balance applications. They would still need to obtain other type of information such as total water storage/ water recharged to water table/change in soil moisture or evapotranspiration for the area (or basin) of interest. The app will provide users with the runoff and rainfall component. Additionally, among the other useful applications of the generated runoff product and app is flood risk assessment. If combined with slope/aspect data, and upslope area, the global runoff data can be used for flood risk assessment. Users can either use the app, the generated runoff collection, or the existing code^40^ which will save them a lot of time and effort if they decide to build on this data for future work. The uncertainty of the GCN250 Runoff product is related to the uncertainty of the GPM rainfall dataset, the SMAP anomalies dataset, and the Curve Number (GCN250) dataset. The accuracy of results is affected by the accuracy of the rainfall data from GPM dataset. The GCN250 app can be further developed into a hydrologic modeling tool where runoff is generated from different equations according users’ choice. For example, different slope-adjustment equations can be added such as equations by Ajmal, *et al*.^42^ or Huang, *et al*.^43^, different CN conversion formulas (from CN_I_ to CN_II_ and CN_III_), different rainfall products can be tested, initial abstraction value can become a user-defined input, and automatic calibration can be applied. Future work can be done on the GCN250 Runoff app using machine learning classifiers, such as random forest, to calibrate the NRCS-CN model used. All the input parameters required to calibrate the model are found in GEE except for the *in-situ* discharge data which can be imported by the user. *In-situ* discharge data need to be present for a prolonged period of record and missing months data need to be removed for better calibration results.

## Data Availability

The application is publicly available and can be accessed via the following link: https://jaafarhadi.users.earthengine.app/view/runoff-from-gcn250. The code for the application^40^ is available here on figshare in a text file (Code.txt): 10.6084/m9.figshare.19596157.

## References

[1] Jaafar HH, Ahmad FA, El Beyrouthy N (2019). GCN250, new global gridded curve numbers for hydrologic modeling and design. Scientific data.

[2] Al-Ghobari H, Dewidar A, Alataway A (2020). Estimation of surface water runoff for a semi-arid area using RS and GIS-based SCS-CN method. Water.

[3] Tiwari K, Goyal R, Sarkar A (2018). GIS-based methodology for identification of suitable locations for rainwater harvesting structures. Water resources management.

[4] Ponce VM, Hawkins RH (1996). Runoff curve number: Has it reached maturity. Journal of hydrologic engineering.

[5] Verma S, Verma R, Mishra S, Singh A, Jayaraj G (2017). A revisit of NRCS-CN inspired models coupled with RS and GIS for runoff estimation. Hydrological Sciences Journal.

[6] Adam, E. O., Abd Elbasit, M. A., Solomon, T. & Ahmed, F. Integration of satellite rainfall data and curve number method for runoff estimation under semi-arid wadi system. *International Archives of the Photogrammetry, Remote Sensing & Spatial Information Sciences* **42**, 10.5194/isprs-archives-XLII-3-W2-1-2017 (2017).

[7] Ajmal M, Waseem M, Ahn J, Kim T (2015). Improved runoff estimation using event-based rainfall-runoff models. Water Resources Management.

[8] Song W, Jiao J, Du P, Liu H (2021). Optimizing the soil conservation service curve number model by accounting for rainfall characteristics: a case study of surface water sources in Beijing. Environmental Monitoring and Assessment.

[9] Krisnayanti DS, Bunganaen W, Frans J, Seran YA, Legono D (2021). Curve Number Estimation for Ungauged Watershed in SemiArid Region. Civil Engineering Journal.

[10] Fan F, Deng Y, Hu X, Weng Q (2013). Estimating composite curve number using an improved SCS-CN method with remotely sensed variables in Guangzhou, China. Remote Sensing.

[11] Deshmukh DS, Chaube UC, Hailu AE, Gudeta DA, Kassa MT (2013). Estimation and comparision of curve numbers based on dynamic land use land cover change, observed rainfall-runoff data and land slope. Journal of Hydrology.

[12] Uwizeyimana D, Mureithi SM, Mvuyekure SM, Karuku G, Kironchi G (2019). Modelling surface runoff using the soil conservation service-curve number method in a drought prone agro-ecological zone in Rwanda. International Soil and Water Conservation Research.

[13] Shammout MaW, Shatanawi M, Nelson J (2018). Curve number applications for restoration the Zarqa River Basin. Sustainability.

[14] Kibler, D. F. *Urban stormwater hydrology*. (American Geophysical Union, 1982).

[15] Weng Q (2020). Urban Runoff Modeling and Prediction..

[16] Yao L, Wei W, Yu Y, Xiao J, Chen L (2018). Rainfall-runoff risk characteristics of urban function zones in Beijing using the SCS-CN model. Journal of Geographical Sciences.

[17] Schneiderman EM (2007). Incorporating variable source area hydrology into a curve‐number‐based watershed model. Hydrological Processes: An International Journal.

[18] Lyon SW, Walter MT, Gérard‐Marchant P, Steenhuis TS (2004). Using a topographic index to distribute variable source area runoff predicted with the SCS curve‐number equation. Hydrological processes.

[19] Youn CH, Pandit A (2012). Estimation of average annual removal efficiencies of wet detention ponds using continuous simulation. Journal of Hydrologic Engineering.

[20] Kumar T, Jhariya D (2017). Identification of rainwater harvesting sites using SCS-CN methodology, remote sensing and Geographical Information System techniques. Geocarto International.

[21] Kadam AK, Kale SS, Pande NN, Pawar N, Sankhua R (2012). Identifying potential rainwater harvesting sites of a semi-arid, basaltic region of Western India, using SCS-CN method. Water resources management.

[22] Singh P, Yaduvanshi B, Patel S, Ray S (2013). SCS-CN based quantification of potential of rooftop catchments and computation of ASRC for rainwater harvesting. Water resources management.

[23] Williams J, Kannan N, Wang X, Santhi C, Arnold J (2012). Evolution of the SCS runoff curve number method and its application to continuous runoff simulation. Journal of Hydrologic Engineering.

[24] Yadupathi Putty MR (2009). Curve-number-based watershed model incorporating quick subsurface runoff, with applications in the Western Ghats, South India. Journal of Hydrologic Engineering.

[25] Mishra SK, Singh VP (2004). Validity and extension of the SCS‐CN method for computing infiltration and rainfall‐excess rates. Hydrological processes.

[26] Jain MK, Durbude DG, Mishra SK (2012). Improved CN-based long-term hydrologic simulation model. Journal of Hydrologic Engineering.

[27] Aragaw, H. M. & Mishra, S. K. Runoff curve number-potential evapotranspiration-duration relationship for selected watersheds in Ethiopia. *Modeling Earth Systems and Environment*, 1–12, 10.1007/s40808-021-01193-6 (2021).

[28] Kannan N, Santhi C, Williams J, Arnold J (2008). Development of a continuous soil moisture accounting procedure for curve number methodology and its behaviour with different evapotranspiration methods. Hydrological Processes: An International Journal.

[29] Mishra S (2014). Relationship between runoff curve number and PET. Journal of Hydrologic Engineering.

[30] Hong, Y., Adler, R. F., Hossain, F., Curtis, S. & Huffman, G. J. A first approach to global runoff simulation using satellite rainfall estimation. *Water Resources Research* **43**, 10.1029/2006WR005739 (2007).

[31] Gorelick N (2017). Google Earth Engine: Planetary-scale geospatial analysis for everyone. Remote sensing of Environment.

[32] Entekhabi D (2010). The soil moisture active passive (SMAP) mission. Proceedings of the IEEE.

[33] Huffman GJ (2015). NASA global precipitation measurement (GPM) integrated multi-satellite retrievals for GPM (IMERG). Algorithm Theoretical Basis Document (ATBD) Version.

[34] Van Zyl JJ (2001). The Shuttle Radar Topography Mission (SRTM): a breakthrough in remote sensing of topography. Acta Astronautica.

[35] Rodell M (2004). The global land data assimilation system. Bulletin of the American Meteorological society.

[36] Muñoz-Sabater J (2021). ERA5-Land: A state-of-the-art global reanalysis dataset for land applications. Earth System Science Data.

[37] Mockus, V. & Hjelmfelt, A. Part 630 hydrology: national engineering handbook Chapter 10 estimation of direct runoff from storm rainfall. (2004).

[38] Sharpley, A. N. & Williams, J. R. EPIC. Erosion/Productivity impact calculator: 1. Model documentation. 2. *User manual*. (1990).

[39] Pfafstetter O (1989). Classification of hydrographic basins: coding methodology. unpublished manuscript, Departamento Nacional de Obras de Saneamento, August.

[40] Jaafar H (2022). figshare.

[41] Moriasi DN, Gitau MW, Pai N, Daggupati P (2015). Hydrologic and water quality models: Performance measures and evaluation criteria. Transactions of the ASABE.

[42] Ajmal M, Waseem M, Ahn J-H, Kim T-W (2016). Runoff estimation using the NRCS slope-adjusted curve number in mountainous watersheds. Journal of Irrigation and Drainage Engineering.

[43] Huang M, Gallichand J, Wang Z, Goulet M (2006). A modification to the Soil Conservation Service curve number method for steep slopes in the Loess Plateau of China. Hydrological Processes: An International Journal.

